# Prognostic Role of L1CAM in Endometrial Cancer

**DOI:** 10.3390/diagnostics15162115

**Published:** 2025-08-21

**Authors:** Mousa Mobarki

**Affiliations:** Department of Basic Medical Sciences (Pathology), Faculty of Medicine, Jazan University, Jazan 45142, Saudi Arabia; dr.mobarki@gmail.com or mamobarki@jazanu.edu.sa

**Keywords:** L1, L1CAM, endometrial, prognosis, molecular, biomarker

## Abstract

The L1 molecule is a cell adhesion molecule (L1CAM) that was originally implicated in neuronal development. In recent years, studies of several large cohorts of patients with endometrial cancer have revealed that L1CAM acts as a poor prognostic factor, in most cases independent of other parameters. It seems to be an important factor, especially in the non-specific molecular profile subgroup (p53 normal expression, MMR proficient, POLE not mutated) of endometrial cancer, and a factor predictive of the response to chemotherapy. This review aims to gather most of the current knowledge regarding this promising prognostic factor.

## 1. Introduction

L1CAM was discovered over 40 years ago as a neural cell adhesion molecule; however, it has only recently gained attention in gynecological pathology for two reasons: as a surrogate marker of TRAF7 mutations in the diagnosis of adenomatoid tumors and well-differentiated papillary mesothelial tumors, and as a prognostic factor in endometrial carcinoma. Several recent studies have shown that L1CAM expression is associated with high-risk endometrial cancer, retains its prognostic significance in multivariate analysis, and might predict response to chemotherapy. This review aims to gather information regarding the pathophysiology of this molecule and its prognostic role in endometrial cancer by presenting the studies that have led to the current state of knowledge.

## 2. L1CAM Physiology and Pathways

In the early 1980s, a cell line called PC12, cloned from a rat adrenal pheochromocytoma, was extensively used to study neuronal differentiation [1]. When this cell line was treated with nerve growth factor (NGF), a marked induction of a large membranous glycoprotein was observed, which, on the basis of these properties, was named NGF-inducible large external (NILE) glycoprotein (GP) [1]. This NILE GP was found in almost all kinds of neuron cultures from the central and peripheral nervous systems [1]. At that time, two well-known cell adhesion molecules were implicated in interactions during nervous system development: the neural cell adhesion molecule (N-CAM), which is expressed at early developmental stages, and the L1 cell adhesion molecule, which appears later in development [2]. Further studies revealed that the L1 molecule and NILE GP were actually the same molecule [2] and that the L1 molecule was implicated in neuron-neuron interaction but not in neuron-astrocyte adhesion, as N-CAM did [3]. Later, it was shown that L1 is a member of the immunoglobulin (Ig)-domain superfamilies, which, along with integrin, cadherin, and selectin families, play important roles in cell–cell interactions during development [4]. This Ig-domain cell adhesion molecule (CAM) superfamily contains several subgroups, such as the L1 and N-CAM families, according to the number and arrangement of their domains [4]. The L1 adhesion molecule, also called CD171, is a 200–220 kDa type I membrane glycoprotein of the immunoglobulin (Ig) family, consisting of six Ig-like domains and five fibronectin-type III repeats, a transmembrane region, and a cytoplasmic tail [5]. Members of the L1 subgroup of CAMs have been implicated in neurons migration, myelination, axonal growth, and pathfinding, and mutations in L1 genes cause severe neurological defects [4]. Despite this major role in the development of the neural system, further studied revealed that L1CAM is involved in many other tissues, such as in kidney morphogenesis [6], that it is present in granulocytes and lymphocytes [7], and in the development of several tumors, such as melanoma [5,8], renal cell cancer [9,10], colorectal cancer [11], gallbladder cancer [12], pancreatic cancer [13], and even gastrointestinal stromal tumors [14]. The monoclonal antibody UJ127, which binds to the extracellular domain of L1CAM, was used in the latter case, showing expression in 74% of the 72 tumors studies [14]. Furthermore, the expression of two monoclonal antibodies was studied by immunohistochemistry in a variety of normal and neoplastic tissues; among normal tissues, only the renal collecting tubules and peripheral nerve bundles showed L1CAM expression [15]. Regarding neoplastic ones, gynecological tumors, some neuroendocrine and neural tumors, as well as melanomas, showed L1CAM expression, whereas most carcinomas of other sites did not express L1CAM [15].

Before presenting the studies that revealed the importance of L1CAM in endometrial cancer, it is worth reiterating that L1CAM is used in the diagnostic pathology of two other neoplasms often encountered in gynecological pathology: the adenomatoid tumor and the well-differentiated papillary mesothelial tumor [16,17,18,19]. These tumors harbor tumor necrosis factor receptor-associated factor 7 (TRAF7) mutations, which are thought to activate nuclear factor-kappa B (NF-κB) signaling and, thus, the expression of L1CAM (clone UJ127.11, dilution 1:1800) by the neoplastic mesothelial cells of these tumors, since L1CAM is considered a transcriptional target of NF-κB [16,17]. However, no further studies exist on the link between TRAF7 and L1CAM expression [18]. In any case, how exactly L1CAM controls its cellular effects—especially in the case of cancer cells, where it seems to offer them increased motility and invasiveness, as well as an epithelial-to-mesenchymal phenotype—remains unclear and warrants further research. Apart from adhesion between cells, L1CAM, when cleaved by several proteases and by further post-translational modifications of its fragments, acquires new and heterogeneous functions [20] that contribute to these effects.

## 3. L1CAM in Endometrial Cancer

### Potential Mechanisms of L1CAM (Figure 1)

The mechanism of how L1CAM is activated/overexpressed and the molecular pathways it activates downstream remain unclear. As previously mentioned, L1CAM “offers” an epithelial-to-mesenchymal transition phenotype to tumor cells, which seems to be regulated by TGFb1 in a Slug-dependent manner [21]. L1CAM at the protein and mRNA levels was also found to be expressed in higher levels in endometriosis tissues than in healthy controls [22]. Despite all this evidence for an implication of the L1CAM molecule in endometrial tissue pathophysiology, very limited information is still available on L1CAM gene regulation in endometrial tissue or its subsequent molecular pathway. In 2010, Pfeifer et al. showed that the L1CAM gene harbors two promoters, both of which are present in endometrial cell lines, that appear to be activated in a cell-type-specific manner [23]. The two promoters were activated by Slug, and one of them by the overexpression of b-catenin [23]. It has been previously shown that b-catenin indeed plays a role in the regulation of L1CAM expression in colon cancer [24], and that in pancreatic cells, the upregulation of L1CAM is Slug-dependent and TGFb1-dependent and promotes tumor cell migration and chemoresistance [25]. In addition, epigenetic mechanisms can regulate the expression of the L1CAM gene, which is localized at the Xq28 chromosome; however, no differences in promoter methylation were found between L1CAM-negative and -positive tumors [26]. Given that a negative prognostic impact of L1CAM expression was also reported in patients with ovarian cancer, single nucleotide polymorphisms (SNPs) of the L1CAM gene were sought in 103 ovarian cancer patients and 104 age-matched controls, finding that the genotype AA of one SNP in intron 1 was associated with ovarian cancer presence [27]. In cancer cell lines, L1CAM promotes not only epithelial-to-mesenchymal transition but also chemotherapy and anoikis resistance [28]. Interestingly, in endometrial cancer cell lines, the presence of FBXW7 mutations affects the protein levels of L1CAM [29]. FBXW7 mutations were found to affect L1CAM gene expression using bioinformatics in The Cancer Genome Atlas (TCGA), confirming a relationship between these two targets [30]. In line with the probable role of L1CAM in the epithelial-to-mesenchymal transition, the expression profile data of 1169 epithelial-to-mesenchymal transition-related genes in endometrial cancer from the TCGA were analyzed in comparison to overall survival and revealed that L1CAM is indeed one of the genes implicated [31]. One of the hallmarks of cancer pathophysiology is the immune response to tumors; it has been found that the so-called tertiary lymphoid structures, specialized ectopic lymphoid formations harboring high endothelial venules, are associated with good prognosis and response to immunomodulatory treatment in several forms of cancer [32,33]. A study of these structures in endometrial cancer revealed that L1CAM is expressed inside the lymphoid structures from follicular dendritic cells independently from its tumor expression, and that this L1CAM-expressing lymphoid structure is an independent prognostic factor [34]. This study used the same previously stained slides of the PORTEC-3 trial, which used the clone 14.10 to define the L1CAM-positive lymphoid structures [35]. Of interest, L1CAM gene expression is associated with RNA methylation, and its expression controls the immunological tumor response [36]. In a bioinformatics analysis of endometrial cancers from the TCGA database, the regulators of RNA methylation and their relationship with prognosis and the immune microenvironment were studied, revealing that L1CAM acts as an RNA methylation-related gene and is implicated in the immune response [36]. In a more recent analysis of endometrial cancer cells, the expression of L1CAM promoted the expression of focal adhesion kinase (FAK) and activation of the FAK–GRB2 (growth factor receptor-bound protein 2)–SOS (Son of Sevenless)–RAS (Rat Sarcoma) pathway [37]; FAK is encoded by the protein tyrosine kinase 2 gene (PTK2), which in glioma cells interacts with L1CAM to allow FAK production [37].

## 4. Diagnostic Role of LICAM

Regarding the diagnostic value of L1CAM, it has been proposed that L1CAM expression, together with IMP3 (insulin-like growth factor-II mRNA-binding protein 3) expression, can accurately distinguish low-grade from high-grade endometrial carcinomas [38]. In line with this notion, another study using all three—L1CAM, IMP3, and progesterone receptor (PR)—improved the diagnostic accuracy in preoperative endometrial samples compared to morphological assessment alone in detecting high-grade carcinomas [39]. Despite these findings, at this moment, this marker has no diagnostic utility in endometrial cancer.

## 5. The Prognostic Role of L1CAM

### 5.1. L1CAM Expression and Endometrial Cancer Histology (Table 1)

One of the first studies regarding L1CAM in gynecological carcinomas was published by Fogel et al. in The Lancet in 2003 [40]. The authors used a monoclonal antibody against the ectodomain of L1 (UJ127.11) and two monoclonal and polyclonal antibodies against the cytoplasmic portion of L1 (polyclonal antibody pcytL1 and monoclonal antibody 745H7) in 72 endometrial carcinomas and 10 non-cancerous hysterectomies [40]. Differences in positivity regarding different antibodies are not reported. They found 20 positive uterine carcinomas, corresponding to 16% of endometrioid adenocarcinomas, 75% of serous carcinomas, and 71% of “mixed” (not further specified) carcinomas; all non-cancerous controls were negative [40]. Higher-stage tumors were all L1CAM positive, compared with 16% of low-stage ones (all histologies included). Soluble L1CAM was also detected in the blood of some patients with L1CAM-positive cancers compared with healthy controls or patients with L1CAM-negative tumors [40]. The authors reported a shorter survival time for L1CAM-positive tumors, but other prognostic factors were not considered in the form of a multivariate analysis [40]. Continuing this research, the authors showed that overexpression of L1CAM in ovarian cell lines enhanced the migration of tumor cells and resulted in better tumor growth in mice [41]. Later, they used two monoclonal antibodies to the ectodomain of L1CAM (L1-11A and L1-14.10) and two to the C-terminal part of L1CAM (2C2 and 745H7), but neither the expression of every different antibody nor the cutoff value for positivity are explained—in 10 normal endometria and in 296 endometrial carcinomas, corresponding to 272 endometrioid adenocarcinomas, 20 serous carcinomas, and 4 clear cell carcinomas [21]. In serous and clear cell carcinomas, the authors described L1CAM expression and ER/PR/E-cadherin negativity (percentages not given) [21]. In the endometrioid group of adenocarcinomas, 29% expressed L1CAM [21]. Patients with L1CAM-positive endometrioid carcinomas showed shorter recurrence-free survival in the univariate analysis, and despite a multivariate analysis mentioned in the discussion [21], these data are not presented to further understand the independent or non-independent role of L1CAM expression. Further techniques in cell lines revealed that TGFb1 enhances L1CAM expression in a Slug-dependent manner and that this upregulation led to a phenotype of epithelial-to-mesenchymal transition, which would be in accordance with their results in the endometrial cancer tissue specimens, where more L1CAM expression was associated with less estrogen receptor (ER), PR, and E-cadherin expression [21].

In 2013, one of the first numerous studies on L1CAM expression in endometrial cancer appeared, corresponding to a retrospective multicenter cohort of 1021 endometrial cancer tissues from stage I endometrioid adenocarcinomas [42]. The study revealed L1CAM expression (clone L1-40.10, cutoff 10%) in 17.7% of cases, and a poorer disease-free and overall survival for these patients [42]. This time, the authors performed a multivariate analysis, where L1CAM expression retained prognostic significance [42]. Slightly later, the Postoperative Radiation Therapy in Endometrial Carcinoma (PORTEC)-1 and -2 trials cohort was used to test L1CAM’s prognostic role [43]. These trials were initially designed to randomize patients with stage I endometrial cancer to receive external beam radiotherapy versus no adjuvant treatment or to receive external beam radiotherapy versus vaginal brachytherapy [43]. Regarding L1CAM, tumor samples of 865 patients were tested with the clone 14.10 (1:500 dilution), and a 10% tumor cell positivity was used as the cutoff value [43]. L1CAM tumor expression was associated with the risk of distant but not vaginal recurrence [43]. It was also associated with overall survival [43]. Most importantly, in multivariate analysis including age, depth of invasion, grade, lymphovascular invasion, and treatment, L1CAM was an independent factor of distant recurrence—a significance retained after excluding non-endometrioid histology [43]. In another study including 86 endometrioid and 30 non-endometrioid carcinomas, when the previously proposed cutoff value of 10% was applied, 44% of the tumors were L1CAM positive (clone 14.10, dilution 1:500) but showed no association with distant metastasis [35]. When the 50% cutoff value was applied, 24% of the tumors were positive and showed a significant association with distant metastasis [35]. Apart from immunohistochemistry, only a few other methods of studying L1CAM in endometrial cancer have been applied. Using the RNA sequencing expression data of 545 uterine carcinomas from TCGA, including all histological types, grades, and stages, Dellinger et al. found that high L1CAM expression (cutoff being the median value) was associated with advanced stage, high grade, serous carcinomas, positive lymph nodes, and poor survival [44]. The prognostic significance of RNA expression was retained in the multivariate analysis [44].

The 10% cutoff value and the 14.10 clone (dilution 1:300) were used in another study of stage I endometrial cancers diagnosed in 388 patients; a 9% positivity was noted, which was not associated with relapse or survival but was associated with relapse in patients not treated with chemotherapy [45]. One of the largest series considering L1CAM expression used the 14.10 antibody (dilution 1:100) and the 10% cutoff value to examine 1199 endometrial carcinomas [46]. The authors found 10% L1CAM expression in stage I endometrioid adenocarcinomas, 18% in 160 advanced-stage endometrioid adenocarcinomas, and 75% expression in non-endometrioid carcinomas [46]. L1CAM expression was significantly associated with advanced stage, positive lymph nodes, high grade and non-endometrioid histology, lymphovascular invasion, and distant recurrences [46]. It was associated with poor survival in endometrioid carcinomas but not in non-endometrioid carcinomas [46]. Another large series of 805 patients was studied for L1CAM expression (tissue microarrays, clone 14.10, dilution 1:300, cutoff 10%) and revealed positivity in 15% of the cases [47]. Similarly to the previous cohorts, L1CAM expression was associated with non-endometrioid histology, advanced stage, positive lymph nodes, lymphovascular invasion, and older age [47]. L1CAM expression was a poor prognostic factor in endometrioid but not non-endometrioid histology—a significance also retained in multivariate analysis [47]. Kommoss et al. reported L1CAM positivity (clone 14.1, dilution 1:50, cutoff 10%) in 8.4% of 344 endometrial carcinomas [48]. In contrast to previous studies, this positivity was not associated with lymphovascular invasion. However, similar to the findings of previous studies, L1CAM expression was an independent poor prognostic factor in the survival analysis [48]. The long-term results of the PORTEC-2 trial, consisting of the 10-year survival of 427 patients treated with external beam radiotherapy with vaginal brachytherapy, revealed L1CAM expression as one of the risk factors associated with pelvic and distant recurrence; multivariate analysis showed that L1CAM was a significant prognostic factor for distant recurrence and overall survival, but not for pelvic recurrence [49]. In another retrospective study of 312 endometrial carcinoma samples, almost 30% of the samples expressed L1CAM, and this expression was associated with distant metastasis but not with disease-free or overall survival [50]. A study of 162 patients with endometrial cancer revealed L1CAM (tissue microarrays, monoclonal antibody, clone UJ127.11, dilution 1:10,000, 10% cutoff) as an independent prognostic factor [51]. In 183 patients with early-stage endometrial cancer, L1CAM expression (clone 14.10, dilution 1:50, 10% cutoff) showed approximately 10% positive cases, and L1CAM was an independent prognostic factor in multivariate analysis [52]. No independent prognostic significance was noted for L1CAM (tissue microarrays, clone 14.10, dilution 1:100, 10% cutoff) in another study of 335 endometrial cancer patients [53].

Given the frequent association of L1CAM with lymph node metastasis, one could wonder if preoperative L1CAM expression can predict lymph node metastasis and prevent unnecessary morbidity of lymph node excision. The study by Zeiter et al. examined the expression of L1CAM in 212 patients and found positivity in 19.3% of the cases with the cutoff value of 10%, but without any association with lymph node metastasis [54]. This study also showed that treatment with radiotherapy improved survival for L1CAM-positive tumors [54]. The expression of L1CAM (tissue microarrays, clone UJ127, dilution 1:30, cutoff 5%) was also studied in the endometrial carcinomas of 34 diabetic patients compared with 34 endometrial carcinomas of matched non-diabetic patients, since the first group is more frequently associated with lymph node metastasis [55]. Despite no difference in L1CAM expression between the two groups, L1CAM expression in endometrial cancer of diabetic patients was associated with pelvic lymph node metastasis [55].

Another large series of L1CAM expression studies considered 1134 curettage specimens, 795 hysterectomy specimens, and the preoperative levels of L1CAM in the blood of 372 patients [56]. The authors found that the level of L1CAM expression in the curettage sample was correlated with that of the hysterectomy one, and that L1CAM expression predicted a poor outcome [56]. L1CAM levels in the blood were also associated with lymph node metastasis and poor outcome [56]. At almost the same time, a similar study compared the L1CAM in 241 endometrial biopsies to that of paired hysterectomy specimens of 75 patients; the serum levels of L1CAM were also measured in 40 patients with endometrial carcinoma [57]. They also showed a concordance between the preoperative biopsies and the hysterectomy specimens, but no association was found between L1CAM serum levels in L1CAM-positive and L1CAM-negative carcinomas [57].

**Table 1:** L1CAM immunohistochemistry in the endometrial carcinoma.

Author, Year	Antibody	Sample Size (*n*)	Positivity	Significance
Fogel et al. (2003) [40]	- L1 ectodomain: monoclonal antibody against the L1 ectodomain (UJ127.11) - L1 cytoplasmic L1 portion: polyclonal antibody (pcytL1) and monoclonal antibody (745H7)	- 72 endometrial carcinomas - 10 non-cancerous Hysterectomy	- 20 carcinomas ○ 16% of endometrioid adenocarcinomas ○ 75% of serous carcinomas ○ 71% of «mixed» (not further specified) carcinomas ○ all non-cancerous controls were negative	- All higher-stage tumors were L1CAM-positive - 16% of the low-stage ones
Huszar M et al. (2010) [21]	- L1CAM ectodomain: two monoclonal antibodies (L1-11A and L1-14.10) - L1CAM C-terminal part: two monoclonal antibodies (2C2 and 745H7)	- 296 endometrial carcinoma cases - 10 normal endometria	- 272 endometrioid adenocarcinomas - 20 serous carcinomas - 4 clear cell carcinomas	- L1CAM-positive endometrioid carcinomas showed shorter recurrence-free survival
Zeimet AG et al. (2013) [42]	- Monoclonal antibody (clone L1-40.10, cutoff 10%)	- 1021 endometrial cancer tissues from stage I endometrioid adenocarcinomas	- L1CAM expression in 17.7% of cases	- Poorer disease-free survival and overall survival in these patients
Bosse T et al., 2014 [43]	- Clone 14.10 (1:500 dilution), cutoff 10%	- 865	- 7%	- L1CAM was an independent factor of distant recurrence
Van Gool IC et al. (2016) [35]	- L1CAM (clone 14.10, dilution 1:500), cutoffs of 10% and 50%	- 86 endometrioid carcinoma - 30 non-endometrioid carcinomas	- 10% cutoff value: 44% of tumors - 50% cutoff value: 24% of tumors	- 10% cutoff value: association with distant metastasis - 50% cutoff value: significant association with distant metastasis
Smogeli E et al. (2016) [45]	- 14.10 clone (dilution 1:300), cutoff 10%	- 388 stage I endometrial cancers	- 9%	- No association with relapse or survival - Association with relapse in patients not treated with chemotherapy
van der Putten LJ et al. (2016) [46]	- 14.10 antibody (dilution 1:100), cutoff 10%	- 1199 endometrial carcinomas	- 10% of stage I endometrioid adenocarcinomas - 18% in 160 advanced-stage endometrioid adenocarcinomas - 75% of non-endometrioid carcinomas	- Significant association with advanced stage, positive lymph nodes, high-grade and non-endometrioid histology, lymphovascular invasion, and distant recurrences - Association with poor survival in endometrioid carcinomas but not in non-endometrioid carcinomas
Pasanen A et al. (2016) [47]	- Clone 14.10, dilution 1:300, cutoff 10%	- 805	- 15%	- Association with non-endometrioid histology, advanced stage, positive lymph nodes, lymphovascular invasion, and older age - Poor prognostic factor in endometrioid but not non-endometrioid histology
Kommoss et al. (2017) [48]	- Clone 14.1, dilution 1:50, cutoff 10%	- 344 patients with endometrial carcinomas	- 8.4%	- No association with lymphovascular invasion - Independent poor prognostic factor in the survival analysis
Wortman BG, Creutzberg CL et al., 2018 [49]	- Clone (not provided), 10% cutoff	- 427 patients treated with vaginal brachytherapy with external beam radiotherapy	- 18 cases - Seventeen cases (multivariable analysis of recurrence in confirmed- high-intermediate risk (HIR) endometrial cancer patients)	- Risk factors associated with pelvic and distant recurrence - Multivariate analysis showed that L1CAM was a significant prognostic factor for distant recurrence and overall survival but not for pelvic recurrence
Klat J et al. 2019 [50]	- L1 antibody (CD171), 1:40 dilution, 10% cutoff	- 312 endometrial carcinomas	- 30%	- Association with distant metastasis - No association with disease-free survival or overall survival
Kim J et al. (2023) [51]	- Monoclonal antibody, clone UJ127.11, dilution 1:10,000, 10% cutoff	- 162 cases of endometrial cancer	- 10%	- The independent prognostic factor
Joe S et al. (2023) [52]	- Clone, 14.10; dilution, 1:50; cutoff, 10%	- 183 early-stage endometrial cancers	- 10%	- Independent prognostic factor in the multivariate analysis
Yoon H et al. (2024) [53]	- Clone 14.10, 1:100 dilution, 10% cutoff	- 335 patients with endometrial cancers	- 10.4%	- No independent prognostic significance
Zeiter D et al. (2021) [54]	- Anti-CD171 (L1) antibody clone 14.10, 1:100 dilution), cutoff 10%	- Preoperative 212 cases	- 19.3%	- No association with lymph node metastasis - Radiotherapy treatment improved survival
Suh DH et al. 2014 [55]	- Clone UJ127, dilution 1:30, cutoff 5%	- 34 endometrial carcinomas in diabetic patients - 34 endometrial carcinomas in non-diabetic patients	- 19.1%	- Association with pelvic lymph node metastasis in diabetic patients
Tangen IL et al. (2017) [56]	- Anti-CD171 (L1) antibody clone 14.10 dilution: 1:100 - The intensity of staining was graded from 0 (no staining) to 3 (strong), and the area was graded as 0, 1 (<10%), 2 (10–50%), and 3 (51–100%)	- 1134 curettage specimens - 795 hysterectomy specimens - 372 preoperative L1CAM blood levels	- 88% (low expression) - 12% (high expression)	- Level of L1CAM expression in the curettage sample correlated with that of the hysterectomy sample - L1CAM expression predicts poor outcome - Blood L1CAM levels were also associated with lymph node metastasis and poor outcome
Pasanen A et al. (2017) [57]	- Monoclonal antibody (CD171; clone 14.10), cutoff 10%	- 241 endometrial biopsy specimens - 75 hysterectomy specimens - 40 serum levels of L1CAM in patients with endometrial carcinoma	- 26.6%	- Concordance between preoperative biopsies and hysterectomy specimens - No association between serum L1CAM levels in L1CAM-positive and L1CAM-negative carcinomas

L1CAM: L1 cell adhesion molecule.

Conclusion: These studies reveal L1CAM expression level in endometrial cancer ranging from 7% to 44%, despite the same two antibody clones and 10% cutoff value almost always being used, raising questions about the preanalytical conditions and the pathologist’s interpretation, which probably impact results. According to the author’s personal experience, finding the optimal immunohistochemical protocol for this antibody is not easy and requires obtaining experience with it. Yet, most of these studies, despite the differences in protocol used or the positivity found, show that higher L1CAM immunohistochemical expression is observed in high-grade histology and advanced stages. Most importantly, the vast majority reveal the poor prognostic impact of L1CAM after adjusting for other factors in multivariate analysis, and they repeatedly show its prognostic significance for stage I endometrioid adenocarcinomas, the most heterogenous group of endometrial tumors lacking sufficient prognostic tools. Thus, despite the observed discrepancies, the fact that higher protein and RNA levels of L1CAM seem to be a poor prognostic factor in most studies highlights this factor as a potentially interesting tool in endometrial pathology.

### 5.2. L1CAM Expression and Molecular Subtype of Endometrial Carcinoma (Table 2)

When the four principal molecular subtypes of endometrial carcinomas started to appear as important prognosticators of this disease, the question of whether L1CAM is just a consequence of one of these subtypes—and therefore not an independent prognostic factor—began to be raised. A study of 947 endometrial carcinomas from patients with early-stage disease originally included in the PORTEC-1 and PORTEC-2 trials was classified according to the four major molecular subtypes: p53-mutant tumors, microsatellite instable tumors, POLE-mutant tumors, and tumors of a non-specific molecular profile. L1CAM expression (clone 14.10, dilution 1:500, 10% cutoff) was associated with distant recurrence and overall survival, and retained significance in multivariate analysis [58]. Karnezis et al. examined 413 endometrial carcinomas previously characterized for their molecular classification and found that 16% expressed L1CAM (tissue microarrays, cutoff 10%) [59]. Its expression was associated with aggressive factors such as advanced stage, non-endometrioid histology, grade 3 endometrioid adenocarcinomas, lymphovascular invasion, and negative ER and PR status [59]. L1CAM expression was associated with a poor outcome [59]. Importantly, it was associated with the p53-mutant tumor group [59]. In the multivariate analysis, L1CAM did not remain a significant prognostic factor [59]. Kommoss et al. further published data on 452 molecularly classified endometrial carcinomas and L1CAM expression (clone 14.10, dilution 1:50, cutoff 10%); they also showed that L1CAM expression was most frequent in p53-mutant tumors [60]. Interestingly, for tumors with no specific molecular profile, L1CAM predicted a poor outcome [60]. Similarly, in a cohort of 94 patients with endometrial cancer, L1CAM expression was of prognostic value only in the non-specific molecular profile subgroup, and in these patients, its expression was associated with early relapse after platinum-based chemotherapy [61]. Pasanen et al. classified 682 endometrioid endometrial adenocarcinomas according to their MMR protein expression (tissue microarrays) and their methylation status [62]. MMR deficiency was associated with a negative L1CAM (clone 14.10, 10% cutoff value for positivity) status. Survival was associated with L1CAM expression in the univariate analysis; however, this was not retained in the multivariate analysis [62]. In a study of 763 patients with endometrial cancer, tumors with abnormal expression of p53, L1CAM (cutoff 10%), ER, and PR showed the worst outcome, but in multivariate analysis, L1CAM retained only marginal significance [63]. The study of prognostic factors in 648 patients with high-risk endometrial cancer failed to show the independent prognostic significance of L1CAM (10% cutoff, clone 14.10, dilution 1:800) [64]. Another large cohort of 1110 non-specific molecular subgroup endometrial carcinomas, gathering data from previously reported cohorts (tissue microarrays, clone 14.10, dilutions 1:25–1:50), showed that L1CAM was a poor prognostic factor in univariate analysis but not in further analyses incorporating the grade of the endometrioid carcinomas and the stage of the disease [65]. L1CAM (clone 14.10, dilution 1:200, cutoff 10%) was studied in 626 stage I endometrioid carcinoma patients, finding expression in 8% of them and no association with survival in the multivariate analysis, but its expression in the non-specific molecular subgroup was associated with poor outcome [66]. In a cohort of 1044 patients with molecularly classified endometrial cancer [67], L1CAM expression (tissue microarrays, clone 14.10, dilution 1:300—according to the previous study by the authors [47], cutoff 10%) was found in almost 15% of the cases, and it was a prognostic factor only in the non-specific molecular subgroup. However, when controlling for further parameters in the multivariate analysis of this subgroup, it did not retain significance [67]. In 486 patients with endometrial cancer whose tumor samples were tested for L1CAM (clone 14.10, dilution 1:200, cutoff 10%), 53% of tumors expressed L1CAM—a higher percentage than most previous studies mentioned above, probably explained by including high-risk patients in this study [68]. L1CAM expression was a poor prognostic factor in both univariate and multivariate analysis, even when including the molecular subtypes [68]. However, in this study, L1CAM did not affect the chemotherapy effect (see discussion below) [68]. In a study of 61 patients with advanced-stage endometrial cancer with wild-type p53 expression and proficient MMR expression, L1CAM expression (clone UJ127.11, dilution 1:1000, cutoff 10%) was a poor prognostic factor [69]. Another study regarding L1CAM expression (technical details not provided) was conducted in 392 low-grade (endometrioid adenocarcinoma, grade 1 and 2) and 183 high-grade (grade 3 endometrioid adenocarcinoma, serous and clear cell carcinoma) cases, showing the independent significance of L1CAM expression in predicting recurrence only in the high-grade group in the multivariate analysis integrating several parameters but not the molecular classification [70]. Endometrioid adenocarcinoma specimens from 142 patients were tested for L1CAM and HER2 expression, revealing 27% and 12% positive cases, respectively [71]. The shortest disease-free survival was noted for patients expressing both HER2 and L1CAM [71]. Van der Putten et al. examined 293 endometrial carcinomas for ER, PR, and L1CAM expression to determine whether their combined study can be of prognostic value [72]. A 10% positivity cutoff value was used for all three markers [72]. They found 18% positivity for L1CAM [72]. L1CAM positivity and ER and PR negativity were associated with advanced stage, non-endometrioid histology, high grade, lymphovascular invasion, and shorter disease-free survival [72]. L1CAM did not retain prognostic significance in the multivariate model, whereas loss of PR did [72]. In a large retrospective study of prognostic algorithms in patients with endometrial cancer, L1CAM was an important risk factor in the p53-mutated subgroup [73].

Conclusion: These studies in molecularly classified endometrial carcinomas show even more variable results than previously noted in non-molecularly classified tumors. Some show that L1CAM remains a poor prognostic factor when adjusting for molecular subtypes, while others fail to find prognostic significance in multivariate analysis. They often show an association with the p53 mutated subtype which is in accordance with the previous finding of association with high grade histology. However, the results are conflicting in the nonspecific subtype; some studies found L1CAM’s poor prognostic role in this subtype, while others did not. The reasons for this discrepancy are not clear but the retrospective nature of these studies gathering tissues from different centers spanning several years, as well as the same issues of different protocols and preanalytical conditions, as previously mentioned, could contribute to the observed differences.

### 5.3. L1CAM Expression and Rare Endometrial Cancer Types (Table 3)

As for rarer histologic types, L1CAM expression was also studied in 90 uterine carcinosarcomas, revealing that, using the 10% cutoff value, 65.4% of the cases were positive—in the epithelial component—much higher than in previous cohorts. In this study, no association between L1CAM and prognosis was found [74]. In 52 endometrial clear cell carcinomas, tissue microarrays were stained for L1CAM (clone not provided, cutoff 50%), showing overexpression in 60% of tumors but with no significant correlation with other factors studied or prognosis [75].

### 5.4. L1CAM Expression in Endometrial Cancer Patients Undergoing Surgery and Adjuvant Chemotherapy

Asano et al. examined the possible prognostic role of L1CAM in 161 patients with endometrial cancer undergoing surgery and adjuvant chemotherapy; they found L1CAM expression (tissue microarrays, clone 14.10, dilution 1:50, H-score with the cutoff set at 35) in almost 30% of the cases [76]. Even in this group, L1CAM was associated with non-endometrioid histology and lymphovascular invasion and was a significant predictor of poor survival—a significance retained in the multivariate analysis [76]. L1CAM seems to predict the response of endometrial cancer to chemotherapy [77]. The authors studied two cohorts of 55 and 93 patients with endometrial cancer treated with surgery and adjuvant platinum-based chemotherapy [77]. Almost half of the patients also received radiotherapy [77]. Tumor samples were tested with the 14.10 clone (dilution 1:100), and the cutoff value was set at 10% [77]. In addition, fresh tissue obtained during surgery from 55 patients was used for RNA extraction; an endometrial carcinoma cell line was further used to test platinum sensitivity [77]. The expression of L1CAM at the gene and protein levels was found to be an independent factor of platinum resistance, and experiments on cell lines confirmed this resistance [77]. This is in concordance with the aforementioned study, which showed that despite L1CAM expression not being associated with relapse or survival, it was indeed associated with relapse only in patients not treated with chemotherapy [45].

## 6. Role of L1CAM in Immunomodulation−Serum L1CAM

Given the frequent reports of L1CAM association with endometrial cancer and more aggressive tumors, as well as the known susceptibility of L1CAM to protease activity, it is worth wondering if a soluble form of L1CAM exists. In a study using ELISA to detect the serum L1CAM level in a few patients with endometrial and ovarian cancer compared to non-cancerous patients, the serum L1CAM level was found to be lower in cancer-bearing patients than in healthy women [78]. On the contrary, another study comparing the serum levels of L1CAM between endometrial cancer patients and non-cancerous patients showed significantly higher levels for the first group [79]. Another study showed that the levels of L1CAM in the blood were associated with lymph node metastasis and poor outcome [56], whereas another one showed no difference in L1CAM serum levels between L1CAM-positive and L1CAM-negative carcinomas [57]. No significant difference in the L1CAM serum levels of endometrial cancer patients compared to healthy controls was found in a study of 45 endometrial carcinoma patients [80]. In 65 patients with endometrial carcinoma, serum L1CAM levels were higher at diagnosis than during follow-up, but no statistically significant augmentation was found in patients upon disease recurrence [81].

These results show that serum levels of L1CAM could be an interesting, minimally interventional method; however, the few studies available show variable results, correlation with tissue expression is often discordant or not studied, and methods or cutoff for this kind of sample are not well defined. The number of studies examining serum L1CAM levels is very poor compared with the numerous studies of L1CAM expression in tissue, but the latter ones are retrospective, while a prospective design would be probably required to adequately study serum L1CAM.

## 7. L1CAM as a Therapeutic Target

The prognostic importance of L1CAM in endometrial cancer suggests that it could be used as a therapeutic target. In small cell lung cancer cell lines, a toxin conjugate with an L1CAM monoclonal antibody successfully inhibited tumor progression [82], suggesting that this pathway could be druggable. Similar results were achieved in carcinoma cell lines and in tumor growth in mice in an earlier study [83]. An anti-L1CAM monoclonal antibody used in mice to treat endometriosis showed that this molecule indeed suppressed endometriosis growth [84]. These preliminary results are promising; however, data are scarce, and more translational studies in endometrial cancer are necessary.

## 8. Conclusions

With almost a decade of research on L1CAM and endometrial carcinoma, the results of its expression in thousands of patients have been published. In almost all these studies, the same 10% cutoff value of tumor positivity and the 14.10 clone, followed by the UJ127.11 one, was used. Most studies revealed an association of L1CAM expression with non-endometrioid, high-grade histology, advanced stages, lymphovascular invasion, and poor prognosis; this prognostic significance is often, but not always, retained in multivariate analyses. In the era of the molecular classification of endometrial carcinoma, L1CAM appears to be expressed more often by p53-mutant tumors and to have an independent prognostic role only in heterogeneous groups with no specific molecular subtype. The underlying mechanisms leading to its overexpression in some tumors and how it induces a more aggressive cancer cell phenotype downstream remain largely unknown, warranting further studies. Whether targeting L1CAM is a therapeutic option also remains unknown, but it is worth investigating in endometrial cancer.

It is worth reiterating that the current review is not a systematic one or a metanalysis, so strict selection criteria or quality grading of the cited studies is applied. Furthermore, as highlighted earlier, there is substantial heterogeneity in the findings across studies, which limits the ability to define the prognostic, predictive, or diagnostic role of LICAM with high confidence.

To conclude, most L1CAM studies in endometrial cancer are retrospective, often spanning long periods with different treatments, and they are based on different immunohistochemical techniques—different automated systems, different antibodies, different cutoffs, and probably pathologist interpretation-based expected biases. These findings could explain the observed discrepancies and highlight the need for prospective, multicenter studies with well-defined criteria to identify the role of L1CAM, in tissue or blood samples. In practice, there are no guidelines on how to use L1CAM in routine practice, but familiarizing oneself with this antibody could be useful in the future.

## Figures and Tables

**Figure 1:**
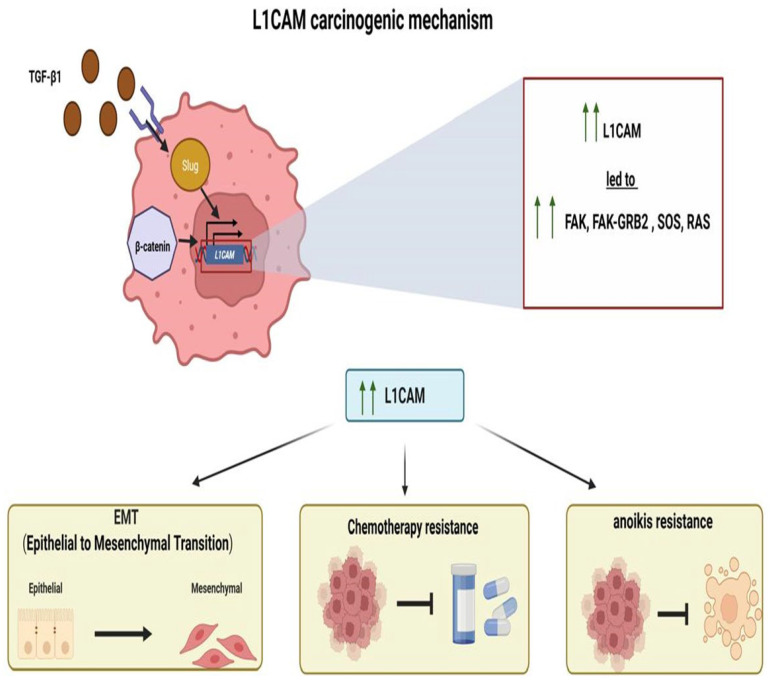
Potential carcinogenic mechanism of L1CAM.

**Table 2:** L1CAM immunohistochemistry in molecularly classified endometrial carcinoma in association with other predictive biomarkers (ER, PR, HER2).

Author, Year	Antibody	Sample Size (*n*)	Positivity	Significance
Stelloo E et al. (2016) [58]	Clone 14.10, dilution 1:500, cutoff 10%	947 early-stage endometrial carcinomas classified molecularly	5.6%	- Association with distant recurrence and overall survival, and retained significance in the multivariate analysis
Karnezis et al. (2017) [59]	Anti-CD171 (L1) Antibody Clone 14.10, cutoff 10%	413 endometrial carcinomas classified molecularly	16%	- Association with aggressive factors such as advanced stage, non-endometrioid histology, grade 3 endometrioid adenocarcinomas, and lymphovascular invasion - Association between negative ER and PR status - Association with a poor outcome - Association with the p53-mutant tumor group
Kommoss FK et al. (2018) [60]	Clone, 14.10; dilution, 1:50; cutoff, 10%	452 endometrial carcinomas classified molecularly	21.5%	- L1CAM expression was most frequent in p53-mutant tumors - For tumors with no specific molecular profile, L1CAM predicted a poor outcome
Ravaggi A et al. (2022) [61]	Clone 14.10, 1:100 dilution, 10% cutoff	94 patients with endometrial cancers	30%	- Prognostic value only in the subgroup with no specific molecular profile - Association of early relapse with platinum-based chemotherapy
Pasanen et al. (2020) [62]	Clone 14.10, 10% cutoff	682 endometrial endometroid adenocarcinomas classified according to their MMR protein expression (tissue microarrays) and methylation status	10.77%	- MMR deficiency was associated with negative L1CAM status in the univariate analysis - Survival was associated with L1CAM expression
Vrede SW et al. (2021) [63]	Anti-CD171, clone 14.10, dilution 1:100, cutoff 10%	763 patients with endometrial cancer, tumors with abnormal expression of p53, ER, and PR	10.4%	- Worst outcome
Vermij L. et al. (2023) [64]	Clone 14.10, dilution 1:800, 10% cutoff,	648 patients with high-risk endometrial cancer	27.8%	- Patients failed to show the independent prognostic significance of L1CAM
Jamieson A et al. (2023) [65]	Clone 14.10, dilutions 1:25–1:50	1110 non-specific molecular subgroup of endometrial carcinomas	10.6%	- Poor prognostic factor in the univariate analysis but not in further analyses incorporating the grade of the endometrioid carcinomas and the stage of the disease
Lindemann K et al. (2024) [66]	Clone, 14.10; dilution, 1:200; cutoff, 10%	626 patients with stage I endometrial endometrioid carcinoma	8%	- No association with survival in multivariate analysis - Its expression in the non-specific molecular subgroup was associated with a poor outcome of the study
Aro K et al. (2024) [67]	Clone 14.10, dilution 1:300, cutoff 10%	1044 patients with molecularly classified endometrial cancer	15%	- Prognostic factor only in the non-specific molecular subgroup of patients - No significance was found in the multivariate analysis of this subgroup
Kleppe A et al. (2025) [68]	Clone, 14.10; dilution, 1:200; cutoff, 10%	486 patients with endometrial cancers	53%	- Poor prognostic factor in both univariate and multivariate analysis, even when molecular subtypes were included - No impact on the effect of chemotherapy
Kim JC et al. (2024) [69]	Clone UJ127.11, dilution 1:1000, cutoff 10%	62 patients with advanced-stage endometrial cancer with wild-type p53 expression and proficient MMR expression	32.26%	- Poor prognostic factor
Li Y et al. (2025) [70]	N/A	- 392 low-grade (endometrioid adenocarcinoma, grade 1 and 2) - 183 high-grade (grade 3 endometrioid adenocarcinoma, serous and clear cell carcinoma)	- 2.55% (low-grade group) - 16.39% (high grade group)	- Independent significance of L1CAM expression in predicting recurrence only in the high-grade group in the multivariate analysis integrating several parameters but not the molecular classification
Abdel Azim S et al. (2017) [71]	Anti-L1 (clone 14.10), cutoff not provided	142 endometrial endometrioid adenocarcinoma tested for L1CAM and HER2	- 27% L1CAM positive - 12% HER2 positive	- The shortest disease-free survival was noted for patients expressing both HER2 and L1CAM
van der Putten LJM et al. (2018) [72]	Anti-CD171 [L1] antibody clone 14.10, dilution 1:100, cutoff 10%	293 endometrial carcinomas for ER, PR, and L1CAM expression	18%	- L1CAM positivity and ER and PR negativity were associated with advanced stage, non-endometrioid histology, high grade, lymphovascular invasion, and shorter disease-free survival

L1CAM: L1 cell adhesion molecule, ER: Estrogen receptor, PR: Progesterone receptor, MMR: Mismatch repair, HER2: Human Epidermal Growth Factor Receptor 2, N/A: Not available.

**Table 3:** L1CAM immunohistochemistry in rare histological endometrial cancer subtypes.

Author, Year	Antibody	Sample Size (*n*)	Positivity	Significance
Versluis M et al. (2018) [74]	Monoclonal antibodies CD171, clone 14.10, 1:500 dilution, cutoff 10%	90 cases of uterine carcinosarcomas	65.4%	No association between L1CAM level and prognosis
Kim SR et al. 2020 [75]	Clone not provided, cutoff 50%	52 endometrial clear cell carcinomas	60%	No important correlation with other factors or prognosis

L1CAM: L1 cell adhesion molecule.
